# Vitamin D Status in Children: Romania’s National Vitamin D Screening Programme in Context of the COVID-19 Pandemic

**DOI:** 10.3390/medsci13030193

**Published:** 2025-09-16

**Authors:** Mădălin-Marius Margan, Alexandru Alexandru, Cristiana-Smaranda Ivan, Estera Boeriu, Sonia Tanasescu, Ada Maria Cârstea, Norberth-Istvan Varga, Roxana Margan, Alexandru Cristian Cindrea, Rodica Anamaria Negrean

**Affiliations:** 1 Department of Functional Sciences, Discipline of Public Health, “Victor Babes” University of Medicine and Pharmacy, Eftimie Murgu Square 2, 300041 Timisoara, Romania; margan.madalin@umft.ro; 2 Center for Translational Research and Systems Medicine, Faculty of Medicine, “Victor Babes” University of Medicine and Pharmacy, Eftimie Murgu Square 2, 300041 Timisoara, Romania; 3 Department of General Medicine, “Victor Babes” University of Medicine and Pharmacy, Eftimie Murgu Square 2, 300041 Timisoara, Romania; alexandru.alexandru@student.umft.ro (A.A.); smaranda.ivan@student.umft.ro (C.-S.I.); 4 Department of Pediatrics, “Victor Babes” University of Medicine and Pharmacy, Eftimie Murgu Square 2, 300041 Timisoara, Romania; tanasescu.sonia@umft.ro (S.T.); carstea.ada@umft.ro (A.M.C.); 5 Department of General Medicine, Doctoral School, “Victor Babes” University of Medicine and Pharmacy, Eftimie Murgu Square 2, 300041 Timisoara, Romania; norberth.varga@umft.ro (N.-I.V.); alexandru.cindrea@umft.ro (A.C.C.); 6 Department of Microbiology, Discipline of Hygiene, “Victor Babes” University of Medicine and Pharmacy, Eftimie Murgu Square 2, 300041 Timisoara, Romania; roxana.margan@umft.ro; 7 Center for Studies in Preventive Medicine, Faculty of Medicine, “Victor Babes” University of Medicine and Pharmacy, Eftimie Murgu Square 2, 300041 Timisoara, Romania; 8 Emergency Clinical Municipal Hospital, 300254 Timisoara, Romania; 9 Department of Preclinical Disciplines, Faculty of Medicine and Pharmacy, University of Oradea, 410073 Oradea, Romania; rodicanegrean@uoradea.ro

**Keywords:** vitamin D deficiency, pediatric screening, national health programme, COVID-19 pandemic, 25-hydroxyvitamin D, healthcare policy, public health, risk-based screening, vitamin D insufficiency

## Abstract

Background and Objectives: Vitamin D deficiency affects bone health and immune function, especially in children. While universal screening is not cost-effective, targeted screening and supplementation strategies have proven effective. This study evaluates the effectiveness of Romania’s National Vitamin D Screening Programme in detecting vitamin D deficiency in paediatric patients, while also accounting for the impact of the COVID-19 pandemic. Materials and Methods: This retrospective observational study assessed the effectiveness of Romania’s National Vitamin D Screening Initiative in detecting vitamin D deficiency among children admitted to the Clinical Emergency Hospital for Children “Louis Țurcanu”, Timișoara, from January 2018 to December 2024. Serum 25-hydroxyvitamin D levels were analysed in 3596 tested patients out of 22,353 total admitted patients, to evaluate trends from before, during, and after the COVID-19 pandemic. Patients aged 0–18 with at least one admission were included, regardless of diagnosis. Patients in ICU, surgical departments, non-Romanian citizens, and those with life-threatening conditions were excluded. Logistic regression analysis was used to assess programme impact and risk factors for vitamin D insufficiency. Results: The study population had a mean age of 5.36 years, with 53.57% male patients. Patient admissions dropped significantly during pandemic years (mean of 2057 annually in 2020–2022 vs. 4045.5 in pre-/post-pandemic years). Vitamin D insufficiency (<20 ng/mL) peaked at 33.3% in 2020 and 32.5% in 2023, with lowest rates in 2019 (17.2%) and 2021 (16.5%). The National Screening Programme implementation resulted in 57.1% higher odds of vitamin D testing in 2023–2024 compared with 2018–2019 (adjusted OR = 1.571, 95% CI: 1.429–1.726, *p* < 0.001), with testing rates increasing from 12.6% to 17.5%. Age emerged as the strongest predictor of vitamin D insufficiency, with each additional year associated with 8–9% increased odds of deficiency. Conclusions: The National Vitamin D Screening Programme significantly enhanced detection of vitamin D insufficiency in paediatric populations, despite pandemic-related disruptions. An optimal testing rate of approximately 17% was identified for balancing detection efficiency with resource utilisation. These findings underscore the need for sustained risk-based screening programmes and public health education initiatives to address vitamin D insufficiency in children, particularly in developing countries with limited healthcare resources.

## 1. Introduction

As medical advancements prioritise prevention, proper nutrition plays a key role in reducing disease risk. Vitamin D is essential for skeletal health, immune function, and chronic disease prevention [1,2]. It regulates calcium and phosphorus metabolism, supporting bone development and preventing rickets in children [3,4,5,6,7]. Beyond skeletal health, emerging evidence suggests that vitamin D may play a protective role in autoimmune conditions, with low levels being associated with higher risks of asthma, type 1 diabetes, and inflammatory bowel disease [8,9,10,11,12].

Importance of vitamin D is also supported by findings showing that it enhances innate and adaptive immune response via regulation of antimicrobial peptides like cathelicidin and defensins [13,14,15,16,17]. It was noted that children with a vitamin D deficiency face a higher risk of severe respiratory infections (e.g., pneumonia and respiratory syncytial virus) with particular importance in the context of the COVID-19 pandemic [18,19,20].

The coronavirus disease 19 (COVID-19) pandemic not only posed the risk of combined respiratory infections but was associated with lockdowns resulting in lowered sun exposure and potentially increasing deficiency rates [21,22,23]. While children generally experience milder COVID-19 symptoms [24,25], the long-term effects of pandemic-induced deficiencies remain insufficiently explored. Due to prolonged confinement and lifestyle changes, children became a high-risk group for deficiency-related health consequences, while raised awareness for the importance of vitamin D may have resulted in self-medicated supplementation.

Sunlight contributes to the primary natural source of vitamin D, through the conversion of 7-dehydrocholesterol to pre-vitamin D3 in the skin. This contribution is often limited by modern lifestyles and sunscreen use, which may or may not balance UV protection with synthesis needs [26,27,28,29,30]. Previous research shows that moderate sun exposure of 10–30 min on the face, arms, and legs, depending on skin type and location, is generally sufficient to meet vitamin D requirements without significantly increasing skin cancer risk [31,32].

Rising deficiency rates have prompted targeted screening programmes for high-risk groups, including children, individuals with darker skin, or those with chronic conditions affecting vitamin D metabolism (e.g., obesity, celiac disease, and chronic kidney disease) [33,34,35,36,37,38,39,40,41,42,43,44,45,46]. Research indicates that universal screening is neither cost-effective nor necessary for the general population, as many individuals maintain sufficient levels through sun exposure and diet [36,37]. For example, Finland’s supplementation strategies have effectively reduced paediatric deficiency without universal screening [47,48,49,50,51,52].

The European perspective on vitamin D deficiency management reveals significant regional challenges that underscore the need for comprehensive screening programmes, particularly in Central and Eastern European countries. An older (2016) epidemiological study has documented that 25(OH)D concentrations of below 20 ng/mL and below 12 ng/mL are observed in 40.4% and 13.0% of the European general population, respectively, [53]. In Romania specifically, national examination programmes for high-risk groups have revealed a vitamin D deficiency prevalence of 39.83%, while population-based studies in adults aged 25–64 years demonstrate an overall deficiency prevalence of 24.8%, with predictors including obesity, female sex, rural residence, lower education level, and lower socioeconomic status (results of the European Health Examination Survey (EHES) 2022) [54].

Similarly, the U.S. Preventive Services Task Force (USPSTF) and other major health organisations recommend a risk-based approach that combines screening with strategic supplementation and public health education [37]. This highlights the international concern surrounding this issue, extending beyond the policies of developing countries.

While official data on adult vitamin D levels are available, for the paediatric population, both the EHES 2021 report [55] and World Health Organization (WHO) Child and adolescent health in Europe 2021 report do not address vitamin D status [56]. The heterogeneous nature of current efforts and guideline recommendations with little focus on vitamin D importance beyond classical health issues, such as rickets prophylaxis, emphasises the urgent need for harmonisation of health status evaluation and public health actions.

Even though several studies look at vitamin D levels in children in Romania [57,58,59,60,61] and in Europe [62,63,64,65,66], with most of them proposing public health measures such as screening or supplementation, evaluation of the said public screening programmes’ effectiveness and impact presents a gap in the literature, with studies only focusing on the impact of supplementation [67,68,69].

This study aims to evaluate the role of Romania’s “National Programme to Assess Vitamin D Status by Serum 25-OH-Vitamin D Measurement of People in Risk Groups” in shaping vitamin D testing patterns and diagnosis rates of deficiency in paediatric populations, while accounting for the epidemiological disruptions caused by the COVID-19 pandemic.

It also measures the expected impact of the COVID-19 pandemic on such programmes, as well as impact on 25-OH-Vitamin D levels and testing patterns. These findings aim to inform evidence-based public health policies and strategies for paediatric populations along with discussions of potential screening strategies, attempting to point at potential risk factors of populations associated with deficiency.

## 2. Methods

### 2.1. Study Design, Population and Data Collection

This study employs a retrospective observational approach, using aggregated historical data, to evaluate the effectiveness of the National Vitamin D Screening Initiative in Romania in detecting vitamin D deficiency in children. The study also examines pre- and post-pandemic data due to the impact of COVID-19 on patient presentation. Data was collected at a single time point, January 2025.

The study timeline, namely January 2018-December 2024, was directly determined in the context of the COVID-19 pandemic in Romania as well as the date of implementation for the screening initiative. The National Programme to Assess Vitamin D Status by Serum 25-OH-Vitamin D Measurement of People in Risk Groups had effective implementation in affiliated state hospitals since 2020. As such, the selected timeline contains significant time before and after the implementation of the National Programme, as well as the beginning and the end of the COVID-19 pandemic.

The study population represented patients with at least one admission in the specified timeframe at Clinical Emergency Hospital for Children “Louis Țurcanu”, Timișoara, representing the largest paediatric hospital in western Romania.

In Romania, all children from birth until the age of 18 months receive vitamin D supplementation as part of the National Rickets Prophylaxis Programme [70], followed by prescribed supplementation. No vitamin D supplementation is administered prior to initial testing as part of clinical practice. Data on parental compliance with post-discharge prophylaxis or individual self-medication with vitamin D preparations were not collected, as this type of assessments are challenging and beyond the scope of this retrospective analysis.

This study was conducted with the approval of the Hospital Ethics Review Committee (no. 408/2025). All procedures involving human participants complied with the principles of the Helsinki Declaration (2013 revision). All procedures were conducted as part of routine clinical practice. Given the retrospective design and complete anonymization of data prior to analysis, specific informed consent for this study was not required.

The study followed the STROBE guidelines [71].

### 2.2. Inclusion and Exclusion Criteria Overview

Inclusion criteria were the following: patients aged 0–18 with at least 1 admission between 1 January 2018–31 December 2024, with no regards of diagnosis or pre-existing conditions. This broad inclusion was intended to offer a better overview of vitamin D status across all paediatric admissions, thereby supporting a more thorough assessment of potential public health implications. The clinical wards of admission of patients included in the study comprised the following clinical wards comprising our hospital: Allergology, Cardiology, Haematology, Nephrology, Ophthalmology, Otorhinolaryngology, Paediatric Oncology, Pneumology, etc.

Exclusion criteria included patients older than 18 years, non-Romanian citizens, and those with a life-threatening condition upon admission. Also, patients discharged upon personal request immediately after admission, regardless of the stated reason, were also excluded from the study. Furthermore, six patients were excluded due to inaccuracies in the database.

To minimise surgical interventional bias, patients admitted to wards with potential related interventions—such as Paediatric Surgery or Orthopaedics—were excluded. Additionally, patients from the Intensive Care Unit were excluded to reduce the potential confounding effects associated with severe, life-threatening conditions.

### 2.3. Sample Collection and Laboratory Assessments

Patient data were collected using the hospital’s electronic record system with censorship of personal data such as parents’ names, addresses, and similar identifiers, only providing demographic and relevant clinical data such as age, gender, underlying diseases, serum 25-hydroxyvitamin D level, etc. Data collection was systematic, utilising Microsoft Excel 2021 (Microsoft Corp., Redmond, WA, USA) for the initial extraction and organisation of demographic, clinical, and surgical variables, ensuring consistency of categorical and continuous data.

Serum 25-hydroxyvitamin D [25(OH)D] levels were measured using the Cobas e 411 analyser (Roche Diagnostics, Mannheim, Germany), which employed an electrochemiluminescence immunoassay (ECLIA) for quantitative detection. The assay is based on a competitive binding principle utilising a ruthenium-labelled 25(OH)D-specific antibody and a biotinylated vitamin D-binding protein.

All analyses were performed in accordance with the manufacturer’s recommendations. Internal quality controls (PreciControl Vitamin D, Roche Diagnostics, Mannheim, Germany) were included to ensure assay reliability. Additionally, external quality assurance was verified using proficiency testing programmes.

Venous blood samples were collected in serum-separating tubes (SSTs) or lithium heparin plasma tubes and centrifuged at 3000× *g* for 10 min at room temperature. The separated serum/plasma was stored at −20 °C or −80 °C depending on the time until analysis. Prior to testing, samples were thawed at room temperature and gently mixed.

Data analysed in this study are generally available in every patient report, with specific data inaccuracies in the database being handled with patient exclusion as mentioned in the Exclusion Criteria Section. The values greater than 140 (upper analytical threshold) were categorised as either potential toxicity or as sufficient depending on the respective analysis, while values lower than 8.1 (lower analytical threshold) were categorised as severe deficiency or insufficient, respectively.

Vitamin D status, based on 25[OH]D levels, was categorised into four groups based on established clinical thresholds: severe deficiency (<10 ng/mL), deficiency (10–20 ng/mL), sufficient (20–50 ng/mL), and potential toxicity (>50 ng/mL), constructed in accordance with both the European Academy of Paediatrics “pragmatic” approach guidelines, as well as the specific American Academy of Pediatrics approach [72,73]. For subsequent analyses, these categories were simplified into two clinically significant groups: “Insufficient” (<20 ng/mL) and “Sufficient” (≥20 ng/mL), to facilitate comparisons and align with standard clinical guidelines for vitamin D status assessment and supplementation.

### 2.4. Statistical Analysis

Descriptive statistics were reported for the overall study population and for subgroups of interest. Graphical methods were used to visualise trends and distributions, including bar charts, line plots, and scatter plots with fitted curves to display patient counts, vitamin D testing patterns, category distributions, and detection trends over time.

To differentiate individuals across multiple admissions during the 7-year period, each patient was identified using a unique anonymized ID. For those with multiple admissions, only one admission per patient was considered for descriptive statistics such as age: the admission closest to the first vitamin D test was selected or the first chronological admission for untested patients. No patient was included more than once.

To differentiate screening from follow-up testing, a correlation between anonymized patient ID, date of admission, and date of testing was performed. The earliest available test date was assigned as the “first-time test” for each patient in the dataset.

The analysis focused on patients who received at least one vitamin D test. Only broad contextual comparisons were conducted for the full hospital population, while detailed statistical analyses were limited to the vitamin D-tested group.

Discharge diagnoses were classified according to ICD-10 chapters. For each patient, the primary discharge diagnosis was mapped to one of the main ICD-10 groups (e.g., infectious diseases, neoplasms, endocrine/metabolic, nervous system, etc.). ICD-10 codes beginning with “R” (symptoms, signs, and abnormal findings) were grouped accordingly. In cases of multiple admissions, more specific categories were prioritised where applicable.

Non-normally distributed variables (e.g., age) were analysed using the Kruskal–Wallis test, followed by Bonferroni-adjusted (ensures better significance levels) Dunn’s post hoc test. For categorical variables, including gender and diagnosis group, Chi-square tests of independence were used to assess distribution differences across years. Spearman’s correlation was used to explore associations between monthly admissions and test outcomes due to non-normality.

To evaluate the impact of the National Vitamin D Screening Programme, we assessed whether the odds of diagnosing vitamin D insufficiency among tested patients differed before and after the implementation of the National Vitamin D Screening Programme. Because of potential pandemic effects on both patient characteristics, as well as screening patterns, data from the years 2020, 2021, and 2022 were removed from programme impact analysis. Due to small sample size in some counties, the analysis was limited to patients from counties that individually contributed more than 5% of the total patient cohort. To avoid issues of perfect separation in logistic regression, diagnostic categories with no outcome variability (i.e., categories where all patients were either tested or untested) were excluded from the analysis.

The first model, which aimed to assess the impact of the National Vitamin D Screening Programme on testing likelihood (comparing the periods 2018–2019 vs. 2023–2024), the following confounders were included patient age (as a continuous variable), gender, county of residence (to account for regional variation), and discharge diagnostic category, grouped by ICD-10 chapters.

Second logistic regression model was used to explore risk factors associated with vitamin D insufficiency (defined as <20 ng/mL). The baseline model adjusted for age, gender, living environment (urban or rural, used as a proxy for socioeconomic status), county of residence, and diagnostic group. An extended model additionally incorporated log-transformed total healthcare spending, computed as the sum of all reimbursed medical costs across admissions for each patient. This transformation was performed to normalise the highly skewed distribution of healthcare costs and address model instability caused by sparse data in certain spending categories. (e.g., a small category with one patient). Model improvement with the inclusion of healthcare spending was evaluated using likelihood ratio tests and changes in the Akaike information criterion (AIC).

To examine the relationship between the testing rate (*x*) and deficiency detection rate over time, a quadratic regression model was applied (*y*): *y* = *a* + *bx* + *cx*^2^. Model assumptions were validated using standard statistical tests. Normality of residuals was assessed using the Shapiro–Wilk, Anderson–Darling, and Kolmogorov–Smirnov tests. Homoscedasticity was evaluated using the Breusch–Pagan and Goldfeld–Quandt tests alongside visual inspection of residual plots. Independence of residuals was examined using the Durbin–Watson test. Model fit and specification were assessed using F-tests, adjusted R^2^, and condition indices to detect multicollinearity.

Python (v. 3.11.4; Python Software Foundation, Wilmington, DE, USA) was used for data processing, modelling, and assumption validation, while JASP (v. 0.19.3, University of Amsterdam, Amsterdam, The Netherlands) supported group comparisons and descriptive analyses.

## 3. Results

The analysis of vitamin D testing trends and test results revealed dynamic patterns in testing frequency and insufficiency rates, reflecting epidemiological events impact, clinical monitoring practices, and temporal changes in patient vitamin D status.

The analysis included, after applying inclusion and exclusion criteria, 22,353 patients, with 42,908 admissions between 2018 and 2024. Out of these patients, the results will focus on the subgroup of patients of 3596 patients (~16%), which present at least one vitamin D testing.

As such, our study presents a tested population of 3596 patients.

### 3.1. Overview of General Study Population Across the Years

Our study included a general study population comprising 22,353 unique patients across the 7-year study timeframe. The mean age of patients was 5.36 years (median 3.0 years). The study population demonstrated a slight male predominance with 56.96% male patients compared with 43.04% female patients. Further yearly details can be found in Table 1.

There were statistically significant differences in age distribution across the years studied (Kruskal–Wallis H test, *p* < 0.001). No significant association between gender distribution and year of admission was found (Chi-square test, *p* = 0.08). These findings suggest an inconsistent demographic profile of the study population in terms of age and sex. Upon pairwise Dunn’s post hoc testing for age, we can safely conclude that the years 2020, 2021, and 2022 are the ones standing out (*p* < 0.001).

Patient volume also fluctuated over the study period with the years 2020, 2021, and 2022 showing lower patient counts. The mean number of patients per year was substantially lower during the COVID-19 pandemic years (2020–2022; mean = 2057) compared with pre- and post-pandemic years (2018–2019, 2023–2024; mean = 4045.5). Although the Mann–Whitney U test indicated a strong trend towards significance (*p* = 0.057), the difference did not reach the conventional threshold of statistical significance (*p* < 0.05).

This can be attributed to healthcare disruptions during the COVID-19 pandemic, resulting in a drop in patient healthcare-seeking behaviour.

### 3.2. Characteristics of Tested Study Population

The tested study population is represented by the 3596 patients (16.09%) who underwent at least one 25(OH)D testing during the entire study period. The patients covered a broad age range, reflecting the paediatric population served. The Mann–Whitney U test revealed a statistically significant difference in age distributions between groups (*p* < 0.001). Gender distribution was balanced, with no statistically significant difference between males and females (*p* = 0.214). Further yearly details can be found in Table 2.

To account for socioeconomic influences, we used living environment (urban/rural) as a proxy for socioeconomic status (SES), given the established correlations between urbanisation and access to healthcare/resources [74]. The tested study population presented a mainly urban living environment of 70.2%.

Additionally, when analysing the county of origin to capture whether any geographic variability was present, as expected, Timiș county was the county of origin for most patients (67.07%), followed by Caraș-Severin (7.68%), Hunedoara (6.70%), Arad (6.70%), Mehedinți (4.92%), and Gorj (1.92%).

**Figure 1:**
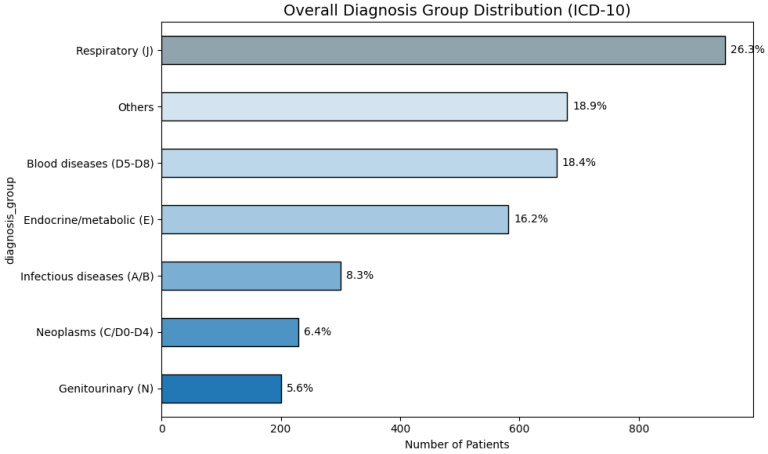
Overview of the most common (>5%) diagnosis groups for the tested patients study group across the seven-year time period.

Among the tested study group patients, the most common primary discharge diagnosis group was “Respiratory (J)” (*n* = 944, 26.25%), followed by “Blood diseases (D5-D8)” (*n* = 662, 18.41%), “Endocrine/metabolic (E)” (*n* = 581, 16.16%) and “Infectious diseases (A/B)” (*n* = 300, 8.34%). “Neoplasms (C/D0–D4)” also accounted for 6.37% (*n* = 229) of patients and “Genitourinary (N)” for 5.56% (*n* = 200). Less frequent categories (<5% each) were combined under “Other” (18.91%). Diagnosis categories distribution for the tested study group is also shown in Figure 1.

There was however, a highly significant difference in the distribution of diagnosis groups across study years (*p* < 0.001). These aspects are presented in detail in the following chapter.

### 3.3. Effect of the COVID-19 Pandemic

When focusing on admission and patient data around the COVID-19 pandemic in Romania, monthly hospital admissions data between 2020 and 2024 revealed distinct patterns coinciding with pandemic-related events and explaining the before mentioned heterogeneity in patient population. These aspects are visible in Figure 1.

In January 2020, admissions were high, reaching above 700 monthly admissions, followed by a precipitous decline. This decline corresponded with the implementation of preventive measures announced by the Romanian government on 22 January, and the general population realising the imminent pandemic danger. By the beginning of March, the first cases were confirmed and the decline in patient admission was further exacerbated following the declaration of a state of emergency, on 16 March 2020.

The period from April 2020 through November 2021 showed consistently suppressed admission levels, generally ranging from 100 up to 200 monthly admissions. A gradual recovery trend began in 2021, but another collapse coincided with Romania’s epidemiological situation in December 2021, being declared the only European Union country in the green zone with a high epidemiological risk and an incidence rate of 1.1 per 1000 inhabitants.

**Figure 2:**
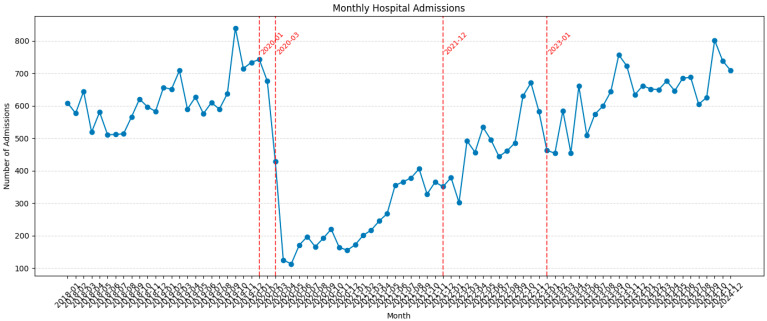
Monthly graph of study years showing pandemic impact on patient admissions with specific timeline markers: 2020-01: implementation of preventive measures announced by the Romanian government; 2020-03: state of emergency declaration; 2021-12: unfavourable epidemiological situation, as declared by European Union; 2023-01: alarming epidemiological situation due to dual COVID-19/influenza infections.

The epidemiological alert declared on 5 January 2023, due to accelerated influenza cases and dual COVID-19/influenza infections, also negatively impacted admission volumes. In this context, the recovery trend plummets again under the 500 monthly admissions mark. The years 2023–2024 demonstrated recovery, with peaks reaching ~800 admissions in 2024, similar to pre-pandemic levels. This is shown in Figure 2.

To further assess the impact of the COVID-19 pandemic on the vitamin D screening, we evaluated, among others, the relationship between the monthly number of vitamin D screened patients and overall hospital activity from 2018 to 2024. Because the distribution of total monthly admissions was non-normal (*p* < 0.001), Spearman’s rank correlation was used to evaluate association. The number of monthly vitamin D screened patients was strongly positively correlated with the total number of monthly hospital admissions (Spearman’s ρ = 0.79, *p* < 0.001), as well as with the number of first admissions per patient (Spearman’s ρ = 0.69, *p* < 0.001).

When considering 2018 and 2019 as pre-pandemic years and 2020, 2021 and 2022, respectively, as pandemic years, a drop of 53.3% in mean annual patients counts between pre-pandemic and pandemic years.

**Figure 3:**
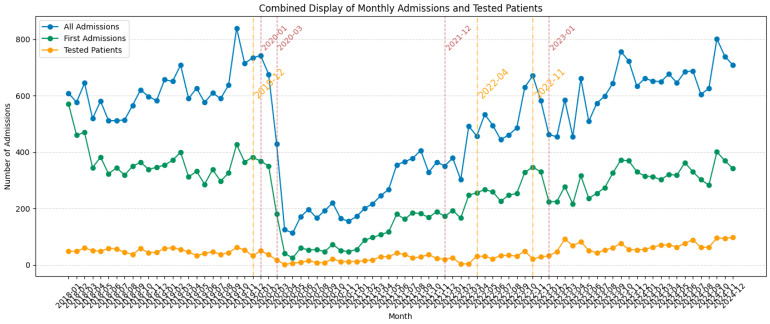
Monthly graph of study years showing impact on total patient admissions (blue), first patient admissions (green), and first-time tested patients (orange); the markers (yellow) for the following programme-related events: 2019-12: approval of National Programme to Assess Vitamin D Status by Romanian government; 2022-04: funding disruptions; 2022-11: support stabilised; pandemic-related events markers (light red) as previously described.

Moreover, The National Screening Programme encountered a significant funding delay during the study period, due to financial resources being oriented towards pandemic-related efforts, though allocations were subsequently restored to baseline levels. A complete overview of admissions, screened patients, and the National Programme-related events can be viewed in Figure 3.

Besides impact on patient presentation counts and funding difficulties, the COVID-19 pandemic impacted both diagnostic profile of patients presenting to our hospital, as well as the testing diagnostic patterns. As previously mentioned, across the study years, there was a significant difference in the distribution of diagnosis groups for both the tested patients’ group and the general patient population (*p* < 0.001).

**Figure 4:**
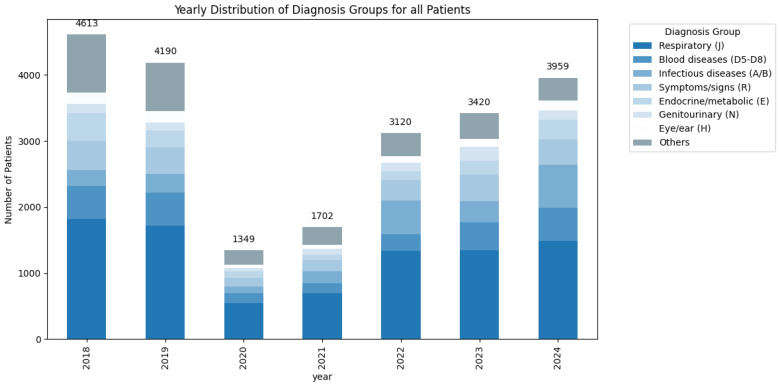
Yearly distribution of admitted patients—stratified diagnosis groups (ICD-10) with absolute patient counts (2018–2024).

For example, although the proportion of infectious disease diagnoses among all patients increased by 7.2% during the pandemic compared with the pre-pandemic period (*p* < 0.001), the absolute number of such admissions declined (from 1504 to 800 cases). Similarly, the proportion of blood diseases decreased by 2.5% (*p* < 0.001), with a reduction in absolute admissions from 1932 pre-pandemic to 549 during the pandemic. For a detailed overview of diagnostic group distributions across years view Figure 4.

**Figure 5:**
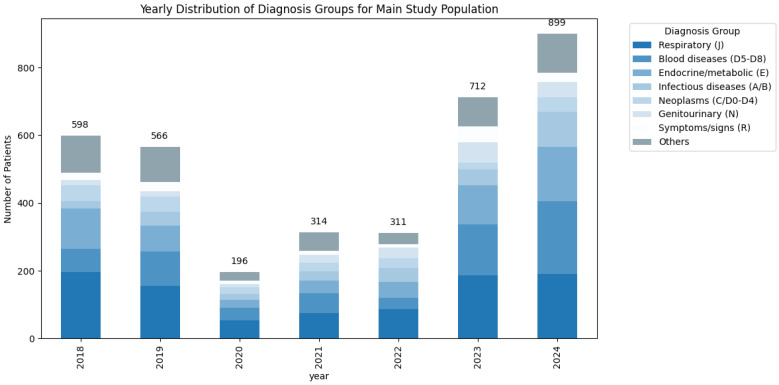
Yearly distribution of tested patients study group—stratified diagnosis groups (ICD-10) with absolute counts of vitamin D-tested patients (2018–2024).

Among vitamin D tested patients, the proportion of neoplasms rose post-pandemic by 6.8% (*p* < 0.001), while endocrine and blood diseases remained proportionally lower during the pandemic period. After the pandemic period, the proportions for these non-infectious diagnoses rebounded toward pre-pandemic levels as hospital utilisation patterns normalised. For a detailed overview of diagnostic group distributions across years for the tested study group refer to Figure 5.

### 3.4. 25-OH-Vitamin D Levels in Tested Study Population

Of the 3596 patients tested for 25(OH)D levels, 1.08% (*n* = 39) of the measurements, had censored results, due to values outside the analytical range. These values were categorised accordingly, resulting in minimal to no impact on analysis.

Detailed categories (severe deficiency < 10 ng/mL, deficiency 10–20 ng/mL, sufficient 20–50 ng/mL, potential toxicity > 50 ng/mL) were initially assessed.

**Figure 6:**
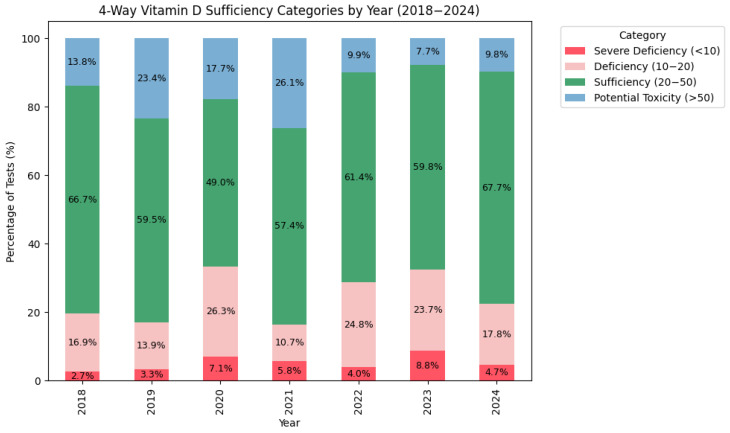
Percentage distribution for the 4 categories (Severe Deficiency/Deficiency/Sufficiency/Potential Toxicity) of 25(OH)D sufficiency by year.

Over the study period, the proportion of children with sufficient vitamin D levels ranged from 49.0% (2020) to 67.7% (2024), while the proportion of those with a severe deficiency varied between 2.7% (2018) and 8.8% (2023). A further breakdown can be seen in Figure 6. The overall distribution in order of proportion was as follows: sufficient (62.2%), deficiency (18.8%), potential toxicity (13.8%), and severe deficiency (5.1%).

**Figure 7:**
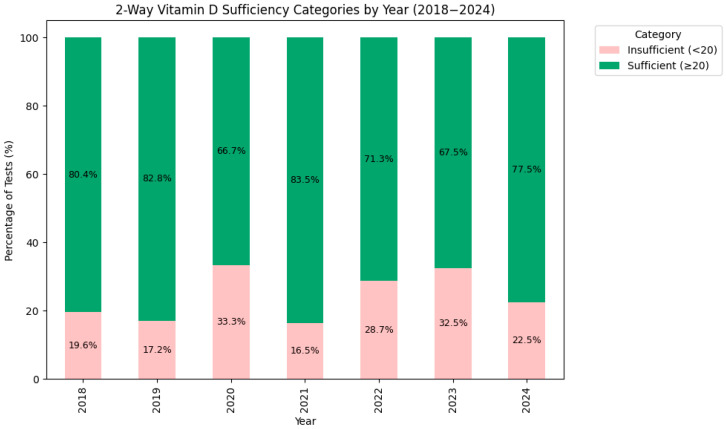
Percentage distribution for the 2 categories (Sufficient/Insufficient) of 25(OH)D sufficiency by year.

For subsequent analysis and to enhance clinical interpretability, the four vitamin D categories were combined into two clinically significant groups: “Insufficient” (<20 ng/mL) and “Sufficient” (≥20 ng/mL), allowing more straightforward comparisons as shown in Figure 7. When looking at the two-way classification, the overall prevalence of the sufficient vitamin D status was 76.1%. The percentage of insufficient patients peaked in 2020 (33.3%) and 2023 (32.5%), while the lowest rates of insufficiency were observed in 2019 (17.2%) and 2021 (16.5%).

### 3.5. Influence of National Programme Implementation

**Figure 8:**
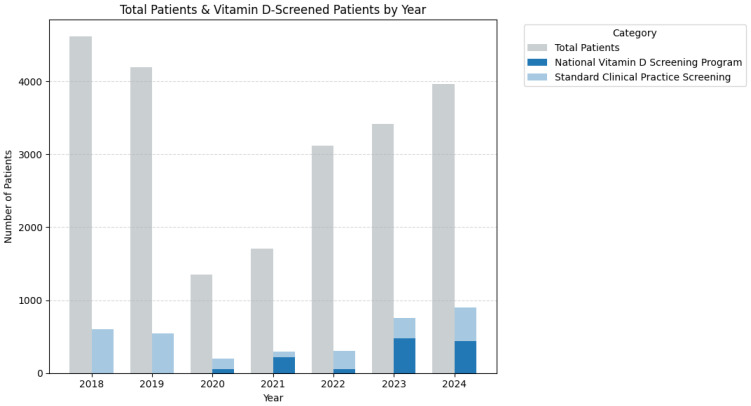
Percentage distribution of the testing in context of patient counts (National Vitamin D Screening Programme or standard clinical practice testing) by year.

The National Vitamin D Screening Programme, implemented effectively in 2020, gradually expanded its coverage even after facing pandemic-related economic challenges, increasing from approximately 200 patients in 2021 to nearly 500 patients by 2024. Figure 8 illustrates the distribution of total patients and vitamin D screening patterns from 2018 to 2024.

**Figure 9:**
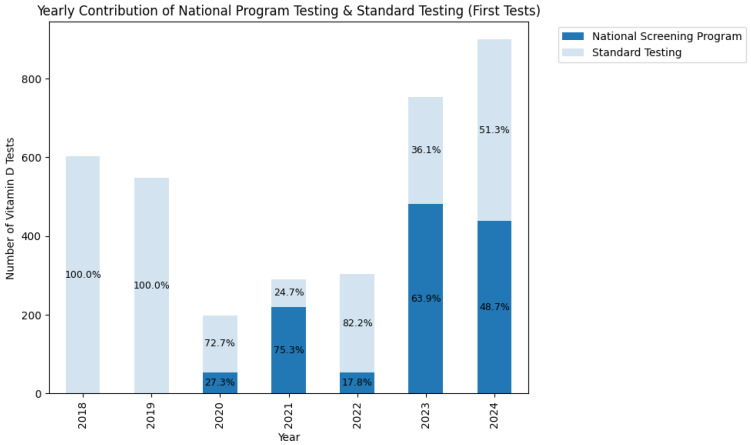
Yearly distribution of first-time vitamin D tests, showing the relative contribution of the National Screening Programme and standard testing from 2018 to 2024.

There was noticeable variation in the use of National Programme testing during the pandemic period with stabilisation in the following years as seen in detail in Figure 9. After initiation in 2020 (~27% screened through Programme), it diverted testing from clinical normal testing practice in 2021 (75%). However, this was followed by a sharp decline in 2022 (~18%) proceeding stabilisation at ~64–49% (2023–2024), suggesting adaptation and funding stabilisation.

As stated previously and illustrated in Figure 8, the testing patterns show significant variation during the pandemic period. Previous analysis has already displayed the magnitude in variation in patient turnover, diagnosis variance, and more. For these reasons, patient data from the pandemic-affected years, namely 2020, 2021, and 2022 were unaccounted for this analysis. This means that of the total 22,353 with 3596 tested, just 16,182 remained (kept 72.4%) with 2775 tested (kept 77.2%).

**Figure 10:**
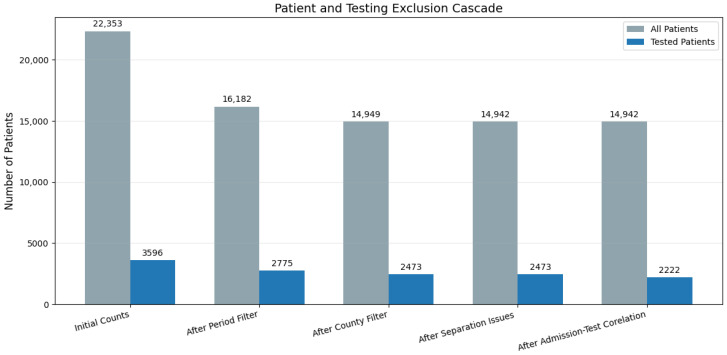
Representation of exclusion steps for logistic regression analysis.

Since many counties contributed to <5% of total patients (very small sample size), only patients from Timiș, Caraș-Severin, Hunedoara, and Arad were included, accounting for a combined 14,949 (66.88% of initial) with 2473 (68.77%) tested. Because of perfect separation issues, another 7 patients had to be removed resulting in 14,942 patients, out of which an unchanged 2473 were tested. However, before running the analysis, another check was performed as for some tested patients their first admission could have been removed during filtering steps, resulting in removal of 251 tested patients from the 2023–2024 period, as their first admission did not match with their testing date. The final breakdown before analysis is as follows: out of the initial 8803, 8030 (91.22%) patients remained and out of the 1164 initially tested, 1011 remained (86.86%), after exclusion for the 2018–2019 period, while out of the initial 7379, 6912 remained (93.67%) with 1611 tested and 1211 (75.17%) post-exclusion. A visual representation of the exclusion cascade is presented in Figure 10.

In other words, the logistic regression analysis included 14,942 patients, with 2222 tested and an overall testing rate of 14.9%. In 2018–2019, 1011 out of 8030 patients were tested (12.59%), while in 2023–2024, 1211 out of 6912 were tested (17.52%), a pre- vs. post-implementation difference of 4.93%.

After adjusting for patient age, gender, county of residence, and diagnostic category, the association between programme implementation and the likelihood of being tested remained significant and strengthened (adjusted OR = 1.571, 95% CI: 1.429–1.726, *p* < 0.001). This suggests that patients in 2023–2024 had 57.1% higher odds of vitamin D testing compared with 2018–2019, independent of demographic and clinical factors. The following patient characteristics were associated with vitamin D testing rates in the adjusted model:

Gender: Male patients had slightly higher odds of testing (OR = 1.119, 95% CI: 1.019–1.228, *p* = 0.018)

Age: Each additional year of age was associated with marginally higher odds of testing (OR = 1.009, 95% CI: 1.000–1.018, *p* = 0.062)

Diagnostic category: Significant variation in testing rates across diagnostic categories, with highest rates among patients with endocrine/metabolic conditions (OR = 1.784 vs. reference category) and lowest rates among patients with external causes of injury (OR = 0.162 vs. reference category).

### 3.6. Exploring Risk Factors for Vitamin D Insufficiency

Another logistic regression was performed, adjusted for demographic and clinical characteristics that could potentially explain a higher prevalence of Insufficiency, in a simplified two-way categorization. The results of the two-step logistic regression analysis to evaluate risk factors for vitamin D deficiency among paediatric patients.

The baseline model included age, gender, environment, diagnosis group, and county. The extended model additionally incorporated log-transformed total healthcare spending.

Age emerged as the most consistent and robust predictor across both models. Each additional year of age was associated with increased odds of vitamin D deficiency (OR ≈ 1.08–1.09; *p* < 0.001), indicating a steady age-related risk.

Gender showed a marginally significant effect, with males having slightly lower odds of deficiency compared with females (OR ≈ 0.80; *p* ≈ 0.04–0.05).

Neither environment (urban vs. rural), nor diagnosis group, nor county were significantly associated with vitamin D deficiency in either model (all *p* > 0.1), suggesting no differential risk by geographic or diagnostic context in this sample.

In the second model, the inclusion of log-transformed healthcare spending significantly improved the model fit (ΔAIC = 17.21, likelihood ratio test *p* < 0.05). Log transformation normalised the highly skewed spending distribution (mean: 2246.29 RON, median: 1308.18 RON). Spending was independently associated with higher odds of deficiency (OR = 1.36 per log unit; 95% CI: 1.186–1.563; *p* < 0.001), implying that individuals with higher healthcare expenditures—often a proxy for greater morbidity—were more likely to be vitamin D deficient.

### 3.7. Exploring the Relationship Between Testing Rates and Vitamin D Insufficiency Detection

**Figure 11:**
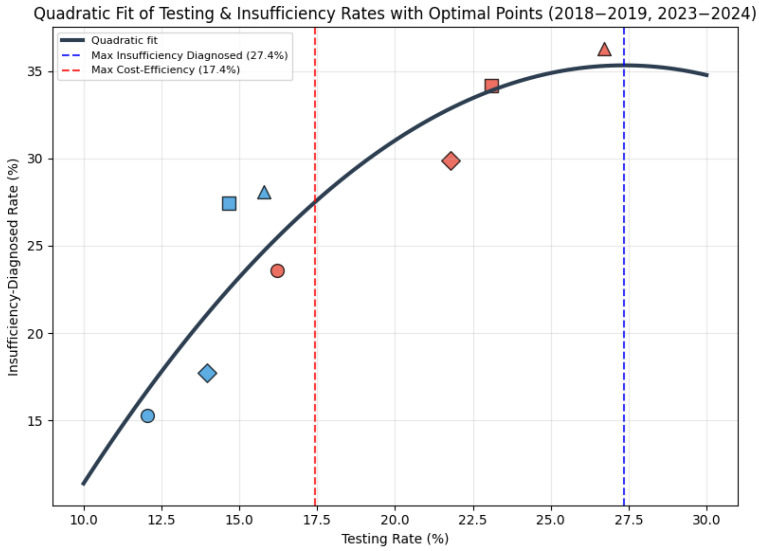
Quadratic relationship between testing and insufficiency rates with main county markers: Timiș—circle, Caraș-Severin—square, Hunedoara—triangle, Arad—diamond. (2018–2019—blue, 2023–2024—red).

To examine the relationship between the proportion of patients tested and the detection rate of vitamin D insufficiency at the first test, a quadratic polynomial model was fitted to the previously filtered data. The analysis yielded a non-linear relationship between the percentage of patients tested and first-time insufficiency rates and can be seen in Figure 11.

The model’s residuals met normality (Shapiro–Wilk *p* = 0.491) but showed slight skewness (0.50) and kurtosis (−1.07). Homoscedasticity was supported (Breusch–Pagan *p* = 0.322), but the Goldfeld–Quandt test (variance ratio = 4.48) suggested potential heteroscedasticity. Independence was confirmed (Durbin–Watson = 1.78), with county residual differences. The quadratic model’s improvement was minimal (F = 1.27, *p* = 0.311), with multicollinearity (condition number = 53.15) and overfitting (training R^2^ = 0.834, CV R^2^ = 0.575). Coefficients were unstable (confidence intervals included zero). The model has moderate reliability, limited by multicollinearity and overfitting.

## 4. Discussion

### 4.1. General Considerations

This cross-sectional study investigates the effect and efficacy of the National Vitamin D Screening Initiative in Romania in detecting vitamin D deficiency in children, while also providing insights into the vitamin D status of Romanian children. This comprehensive analysis examines a paediatric population that spans a seven-year period (2018–2024) revealing insights into how external factors, particularly the COVID-19 pandemic, influenced healthcare. Our study demonstrates that vitamin D testing, while representing only 16% of the total patient population, followed distinct patterns that reflected broader healthcare system dynamics and epidemiological pressures.

The general study population of 22,353 paediatric patients treated between 2018 and 2024 exhibited a relatively consistent demographic profile, with a mean age of 5.36 years and a slight male predominance (56.96% male). While significant differences in age distribution were observed across study years (*p* < 0.001), particularly during the pandemic years 2020–2022, no significant association was found between gender distribution and year of admission (*p* = 0.08). This demographic stability in gender distribution minimises potential confounding effects in our analyses of vitamin D testing patterns and programme effectiveness. The age variations observed during the pandemic period likely reflect the complex interplay of healthcare access barriers, disease presentation patterns, and clinical prioritisation during crisis periods rather than fundamental demographic shifts in the underlying population.

The tested patients study population of 3596 patients who underwent vitamin D testing demonstrated a similar demographic profile but with a significantly different age distribution compared with the general population (*p* < 0.001), with a mean age of 5.78 years. This age differential suggests that clinicians may preferentially order vitamin D testing in older paediatric patients, possibly reflecting clinical guidelines, perceived risk factors, and diagnostic considerations. The consistency in gender distribution between tested and non-tested populations (*p* = 0.214) indicates that gender bias in vitamin D testing decisions was not a significant factor in clinical practice during the study period.

These can be explained by the fact that vitamin D testing is recommended for patients considered at risk, through clinical consideration, rather than general screening practices [75].

### 4.2. Impact of the COVID-19 Pandemic

The most striking finding of our study is the profound impact of the COVID-19 pandemic on patient presentation patterns and vitamin D screening practices. The 53.3% reduction in mean annual patient counts between pre-pandemic (2018–2019) and pandemic years (2020–2022) aligns with global reports of healthcare service disruptions during this period. This substantial decline cannot be attributed solely to reduced disease burden but rather reflects the complex interplay of healthcare system reorganisation, patient behavioural changes, and policy responses to the pandemic. Several studies have reported similar drops in admissions, while also showing a paradoxical drop in acute respiratory diseases [76,77,78,79,80]. This drop in patient presentations can be explained through the lockdown and restriction measures or patients postponing medical care when possible.

The National Vitamin D Screening Programme faced implementation challenges due to a temporary reduction in screening activities, driven by governmental prioritisation of COVID-19 response measures such as testing, hospital capacity, and vaccination efforts. Unfortunately, the identification of Romania as the only European Union country in the green zone with a high epidemiological risk in December 2021 coincided with another collapse in admissions. This finding underscores how local epidemiological conditions can significantly influence healthcare-seeking behaviour and system capacity, even when broader pandemic pressures may be subsiding elsewhere.

Despite this, the programme showed resilience, recovering strongly in 2023–2024 with restored funding as pandemic pressures eased, though the six waves likely impacted population health and healthcare systems variably [81].

Besides effects on healthcare system, clinical findings are also noteworthy. The paradoxical increase in the proportion of infectious disease diagnoses (7.2% increase) despite an absolute decrease in such admissions (from 1504 to 800 cases) suggests that patients with infectious diseases maintained relatively higher healthcare-seeking behaviour compared with those with other conditions. This pattern may reflect the heightened awareness and concern regarding infectious diseases during the pandemic period.

### 4.3. Vitamin D Status and Clinical Relevance

The vitamin D status results reveal important insights into paediatric nutritional health in Romania. The overall prevalence of sufficient vitamin D levels (≥20 ng/mL) was 76.1%, which compares favourably to international paediatric populations but still indicates that nearly a quarter of tested children had insufficient levels.

Using the classical four-category classification of vitamin D levels, sufficiency (>20 ng/mL) remained relatively stable across most years, though notable fluctuations occurred. Severe deficiency peaked at approximately 8.8% in 2023, potentially linked to delayed healthcare access during the pandemic’s aftermath, as paediatric visits remained below pre-pandemic levels. The ~3–9% severe deficiency rate suggests that a subgroup of children remains at risk for adverse health consequences, such as rickets or respiratory infections [20].

This increase likely reflects lockdown measures, which reduced outdoor activities and sun exposure—a primary source of vitamin D synthesis [6,82]. Similar trends were reported in studies from Guangzhou, China, and Italy, attributing increased deficiency to prolonged home confinement [83,84].

However, the 2–7% severe deficiency rate suggests that a subgroup of children remains at risk for adverse health consequences, such as rickets or respiratory infections [20]. This concern is particularly relevant in rural areas, where access to supplementation and preventive care may be limited. Similar patterns have been observed in developing countries, where vitamin D deficiency is associated with increased risk and severity of pneumonia [85,86,87].

The modest variation in deficiency was without a clear trend, while simultaneously, the severe deficiency category presented small numbers of patients (16 in 2018), underlining the shortcomings of this type of classification and analysis.

A simplified two-category model (sufficient vs. insufficient) revealed clear fluctuations in vitamin D status, directly linked to pandemic-related disruptions. Insufficiency rates among first-time testers increased sharply from 19.6% in 2018 and 17.2% in 2019 to 33.3% in 2020, coinciding with widespread lockdowns that reduced outdoor activities—a primary source of vitamin D [26,27,46,88,89]. The literature findings showed that acute infections dropped, leading to a potentially more chronically ill testing population, explaining the difference in percentages. However, that does not seem to be supported by our findings, as respiratory infection admissions seemed to slightly increase. This can be caused by parents being worried about potential complicated or severe respiratory infections and seeking hospitalised medical attention for infections that could have otherwise been treated at home or in ambulatory care.

The most noticeable change is the temporary dip to ~17% insufficiency in 2021, which can be reflect by heightened public awareness of vitamin D’s immune benefits during the pandemic, potentially driving supplementation, as media campaigns emphasised its role in infection prevention [18,24,90,91,92,93,94,95,96,97,98]. However, the resurgence of insufficiency in 2022 (28.7%) and 2023 (32.5%), before a slight decline to 26.78% in 2024, suggests long run effects, possibly due to tendencies in limiting social contact, lingering effects of reduced healthcare access (e.g., only ~1300 patients in 2020 vs. ~4200 in 2019), and even large-scale behavioural shifts, such as prolonged indoor time due to school closures during pandemic lockdown followed by parental caution post-lockdown [83]. These trends underscore the pandemic’s lasting impact on paediatric health status beyond the scopes of just clinical disease diagnosis, pleading to address environmental and behavioural barriers for adequate vitamin D synthesis and supplementation. Even though increased public awareness leading to higher rates of “at home” supplementation [99,100], which in turn lead to the alleviation of insufficiency in 2021, the phenomenon did not last.

For instance, Esposito et al. (2021) [6] reported a 15–20% increase in deficiency among European children during the pandemic, while deficiency rates of 20–40% have been reported in high-risk populations across Northern Europe [49].

Arguments are met for the necessity for permanent public health programmes that educate the general population on the importance of vitamin D status, similar to existing ones on sugar, salt or fat intake, especially in developing countries [101,102,103]. In this context, financial burden may come to mind, yet feasible solutions such as a revised scholar hygiene-education curriculum to address aspects such as vitamin D status, could be implemented.

### 4.4. National Screening Programme

The implementation of the “National Programme to Assess Vitamin D Status by Serum 25-OH-Vitamin D Measurement of People in Risk Groups” was followed by an unprecedented pandemic context which affected its implementation. In 2020, only 27.3% of first-time tests were conducted under the programme, but this rose sharply to 75.3% in 2021 as programme implementation was fully known, not for long, however, due to pandemic financial restrains. Despite fluctuations, the programme maintained a significant role until pandemic situation stabilisation in 2023 and 2024, accounting to ~50–60% of the first test of patients. This upward trend may also be reflected by improved identification of at-risk category children, who would benefit the most of vitamin D testing.

The substantial increase in testing rates achieved by the programme represents more than just improved surveillance—it reflects enhanced healthcare system capacity to identify and address nutritional deficiencies [104]. While official data on adult vitamin D levels are increasingly available across European countries, the lack of systematic paediatric monitoring represents a significant missed opportunity for early intervention and prevention of long-term health consequences.

The observed success of Romania’s targeted screening programme raises important considerations for integrating vitamin D assessment into routine paediatric health evaluations. Given the already documented risk factors—including obesity, rural residence, lower socioeconomic status, and specific medical conditions—there is a compelling case for incorporating vitamin D screening as a standard component of paediatric health assessments when these risk factors are present [55,58,60]. This approach would transform vitamin D screening from an isolated intervention into an integrated element of comprehensive paediatric care.

Such an approach aligns with the broader trend toward preventive medicine and early intervention strategies. When healthcare providers routinely assess children for established risk factors—including dietary limitations, reduced sun exposure, chronic medical conditions, and socioeconomic challenges—vitamin D screening becomes a logical extension of comprehensive health evaluation rather than an additional burden on healthcare systems.

For developing countries, but not limited to, considering similar initiatives, this model suggests that even modest investments in targeted screening programmes can yield significant improvements in the detection of population health condition, particularly when programmes are designed with flexibility to adapt to changing circumstances while maintaining focus on high-risk populations.

To summarise the impact of the National Programme initiative, there were significant increases in vitamin D testing rates following the implementation of the National Vitamin D Screening Programme. After controlling for patient demographics and clinical characteristics, the odds of vitamin D testing increased by 57.1% in the post-programme period compared with the pre-programme period. This represents substantial improvement in screening coverage, with the testing rate increasing from approximately one in eight patients (12.6%) to nearly one in six patients (17.5%).

### 4.5. Optimal Testing Rates and Economic Considerations

Economic considerations are important, particularly in developing countries. A testing rate of approximately 17.4% appears optimal for diagnosing the most insufficient patients with minimal costs, considering that one 25(OH)D serum testing costs about EUR 18. This should be considered as a possible burden especially in developing countries.

Although Romania’s risk-based screening approach likely aimed to prioritise vulnerable populations, the pandemic disruptions made identifying such patients more challenging. Policies such as Finland’s supplementation model, which reduced deficiency to 5% without universal screening [48], might be worth considering along with Romania’s risk-screening strategy.

The exclusion of counties with fewer than 5% of total patients from the regression analysis, while methodologically sound, may have implications for understanding healthcare equity across different geographic regions leading to persisting disparities persist—driven by heterogeneous healthcare access especially in context of pandemic-related disruptions—mirroring systemic challenges seen in other healthcare systems even of developed countries [105]. Tools such as data envelopment analysis could help quantify and address these inefficiencies [106].

Meanwhile, the proportion of patients with potential toxicity rate (~8–26%) observed throughout the study period suggests that vitamin D levels may exceeded so-called “safe thresholds”, indicating a possible likelihood of unsupervised or excessive supplementation, or the need for reconsideration of toxic threshold [107,108,109].

### 4.6. Strengths and Limitations of the Study

This study has several limitations that should be considered when interpreting the findings. First, its retrospective, cross-sectional design limits the ability to establish causality, permitting only the identification of associations between vitamin D status, pandemic-related disruptions, and hospital admissions. Second, potential bias exists as asymptomatic deficiency and limited healthcare access, especially in the pandemic years may lead to this population as being underrepresented. Third, the variation in testing rates across years, notably the decline during 2020–2021 and subsequent increase post 2022, complicates year-to-year comparisons and may have introduced temporal bias. Fourth, data on individual vitamin D supplementation practices were not available, preventing the assessment of the impact of public awareness campaigns and at-home supplementation on vitamin D levels.

The small sample size in number of years (not patients) for regression analysis (after excluding 2020, 2021, and 2022) and the shortcomings of the quadratic model limits statistical power (multicollinearity and overfitting). The exclusion of 2020, 2021, and 2022 from regression analyses, while necessary due to pandemic impact and programme setbacks, may have biased the results if those years had different patterns. Finally, confounders measured by proxies such as severity of medical conditions—healthcare services costs, socioeconomic status—living environment, and confounders unaccounted for such as BMI not being largely available in the database, mostly recorded in patients’ clinical examination records, limit the power of this analysis.

With minimal impact on analysis is the aspect of censored data. By this, we mean that a small proportion of vitamin D measurements (1.08%) were outside the analytical measurement range of the laboratory equipment, resulting in categorisation as described in the methodology.

Another limitation of this study is the lack of data on parental compliance with vitamin D prophylaxis post-discharge of neonatal and infant patients. This aspect is challenging to assess and warrants further research.

Despite these limitations, the study possesses several notable strengths. It includes a large cohort (*n* = 22,353 total patients, 3596 tested patients, with 14,942 and 2222, respectively, accounted for, in the logistic regression model) spanning a 7-year period (2018–2024), enhancing the relevance of the findings to general patient population. The consistent demographic profile across study years minimises bias related to age and sex. Furthermore, the study captures the full trajectory of vitamin D status before, during, and after the COVID-19 pandemic, providing a unique longitudinal perspective on the impact of public health disruptions and interventions. Finally, the study highlights real-world challenges and successes of the national risk-based screening programme, contributing valuable insights for public health strategies aimed at optimising paediatric vitamin D status.

This is also the first study in Romania to evaluate large-scale vitamin D testing (as of the 2019 programme initiative). Also, as of 2021, there were no studies evaluating the impact of vitamin D screening programmes, recorded in the 51 countries categorised as very high on the 2016 Human Development Index [110] as presented by Leila C. et al. in their Evidence Review for the U.S. Preventive Services Task Force [111].

### 4.7. Future Directions

Future public health policies should prioritise optimising vitamin D screening to achieve an efficient and sustained screening rate of approximately 17%, focused on risk group patients, aligning with the identified optimal testing threshold that maximises the detection of insufficiency while minimising costs (~EUR 18 per 25(OH)D serum test). This approach could involve implementing targeted screening protocols in primary care settings, particularly in underserved rural areas where access to healthcare remains limited, ensuring equitable resource allocation and reducing economic burdens in developing countries. Prospective studies evaluating long-term health outcomes of children identified with insufficiency through such optimised programmes are essential to validate their cost-effectiveness and inform policy adjustments.

Additionally, policies should integrate individual-level data collection on supplementation practices, sun exposure habits, dietary intake, and socioeconomic status to refine risk-stratification models, enabling tailored interventions that enhance vitamin D sufficiency. Standardised year-round sampling to assess seasonal variations could guide the timing and frequency of screening campaigns, optimising resource use. Randomised controlled trials combining risk-based screening at the 17% rate with targeted supplementation could provide robust evidence for national guidelines, balancing clinical benefits with economic feasibility.

Moreover, randomised controlled trials assessing the effectiveness of combining risk-based screening strategies with targeted supplementation initiatives could offer valuable insights for optimising paediatric vitamin D guidelines. Finally, public health interventions that integrate sustained educational programmes about vitamin D alongside systematic screening should be prioritised, particularly in developing countries, to ensure long-term improvements in paediatric health outcomes.

The lack in current paediatric health assessment regarding vitamin D, with no evaluation of already implemented public health measures needs further attention, with nationwide measures evaluation, followed by potential secondary analysis such as meta-analyses to show best practice solution applicable internationally.

## 5. Conclusions

This study provides one of the largest single-centre analyses to date of vitamin D testing and status in a paediatric population, comprising 22,353 patients and 42,908 admissions over a seven-year period with focus on the 3596 children who underwent at least one vitamin D test. We were able to characterise demographic, diagnostic, and temporal factors influencing both testing practices and vitamin D insufficiency rates in real-world hospital care.

Our findings show that testing rates and insufficiency prevalence were strongly influenced by healthcare system dynamics, including the COVID-19 pandemic and the implementation of a National Vitamin D Screening Programme. The pandemic was not only associated with a substantial reduction in hospital admissions and testing but also with diagnostic shifts, while the introduction of the screening programme was independently associated with a significant increase in testing likelihood (adjusted OR 1.57, 95% CI 1.43–1.73).

In terms of vitamin D status, 23.9% of tested children were insufficient (<20 ng/mL), with peaks during pandemic-affected years. This prevalence aligns with, though is somewhat lower than, the reported adult prevalence of 24.8–40.4% across European populations, suggesting that paediatric patients require different screening strategies due to different burden and distribution of deficiency. Logistic regression identified age and healthcare spending as consistent predictors of insufficiency, indicating that older children and those with greater morbidity were at particular risk.

From a methodological perspective, our study illustrates the challenges inherent in categorising vitamin D status due to the lack of international consensus on thresholds. We applied a combined framework that harmonises the pragmatic European and the more detailed American Academy of Pediatrics cutoffs, thereby offering a clinically interpretable approach that can be compared across studies. By demonstrating the programme’s ability to increase testing coverage, even despite disruptions, our findings address a global gap in the systematic assessment of paediatric screening initiatives, where research has predominantly focused on supplementation or adult populations.

Taken together, these results highlight the need for more standardised, internationally accepted guidelines for the screening, categorization, and treatment of vitamin D deficiencies in paediatric populations. By documenting both the opportunities and the challenges of large-scale monitoring programmes in a real-world setting, our study contributes empirical evidence that may help guide future public health strategies and inform the development of consensus-driven, evidence-based international recommendations.

The main recommendation of this study is the proposal of integrated testing guidelines, with public health measures to ensure continuity during crises, and prioritising interventions for younger and rural patients to address disparities, while advocating for further longitudinal research to evaluate the impact of screening and supplementation on clinical outcomes, thereby enhancing early deficiency detection and supporting equitable paediatric public health strategies.

## Figures and Tables

**Table 1:** Demographic characteristics by year and patient count for general study population.

Year	Patient Count	Mean Age	Median Age	Male (%)	Female (%)
2018	4613	5.91	4.0	53.96	46.04
2019	4190	5.52	4.0	53.17	46.83
2020	1349	4.99	3.0	54.63	45.37
2021	1702	4.50	2.0	54.76	45.24
2022	3120	4.48	3.0	55.42	44.55
2023	3420	5.39	4.0	51.49	48.51
2024	3959	5.68	4.0	52.99	47.01
Total	22,353	5.36	3.0	53.57	46.43

**Table 2:** Demographic characteristics by year and patient count of tested study group.

Year	Patient Count	Mean Age	Median Age	Male (%)	Female (%)
2018	603	5.49	3.0	52.7	47.3
2019	548	5.55	3.5	58.2	41.8
2020	198	5.74	4.0	61.1	38.9
2021	291	5.32	4.0	56.7	43.3
2022	303	5.49	4.0	53.1	46.9
2023	754	5.76	4.0	54.1	45.9
2024	899	6.38	5.0	54.9	45.1
Total	3596	5.78	4.0	55.2	44.8

## Data Availability

The data presented in this study are available on request. The data are not publicly available due to ethical restrictions and the sensitive nature of patient medical records, in accordance with institutional and national data protection regulations.

## References

[1] Álvarez-Mercado A.I., Mesa M.D., Gil Á. (2023). Vitamin D: Role in chronic and acute diseases. Encyclopedia of Human Nutrition.

[2] Gombart A.F., Pierre A., Maggini S. (2020). A Review of Micronutrients and the Immune System–Working in Harmony to Reduce the Risk of Infection. Nutrients.

[3] Khazai N., Judd S.E., Tangpricha V. (2008). Calcium and vitamin D: Skeletal and extraskeletal health. Curr. Rheumatol. Rep..

[4] Christakos S. (2021). Vitamin D: A Critical Regulator of Intestinal Physiology. JBMR Plus.

[5] Fleet J.C. (2022). Vitamin D-Mediated Regulation of Intestinal Calcium Absorption. Nutrients.

[6] Esposito S., Leonardi A., Lanciotti L., Cofini M., Muzi G., Penta L. (2019). Vitamin D and growth hormone in children: A review of the current scientific knowledge. J. Transl. Med..

[7] Baroncelli G.I., Comberiati P., Aversa T., Baronio F., Cassio A., Chiarito M., Cosci O Di Coscio M., De Sanctis L., Di Iorgi N., Faienza M.F. (2024). Diagnosis, treatment, and management of rickets: A position statement from the Bone and Mineral Metabolism Group of the Italian Society of Pediatric Endocrinology and Diabetology. Front. Endocrinol..

[8] Meeker S. (2016). Protective links between vitamin D, inflammatory bowel disease and colon cancer. World J. Gastroenterol..

[9] Yu J., Sharma P., Girgis C.M., Gunton J.E. (2022). Vitamin D and Beta Cells in Type 1 Diabetes: A Systematic Review. Int. J. Mol. Sci..

[10] Zhu Y., Jing D., Liang H., Li D., Chang Q., Shen M., Pan P., Liu H., Zhang Y. (2022). Vitamin D status and asthma, lung function, and hospitalization among British adults. Front. Nutr..

[11] Alkhatatbeh M.J., Almomani H.S., Abdul-Razzak K.K., Samrah S. (2021). Association of asthma with low serum vitamin D and its related musculoskeletal and psychological symptoms in adults: A case-control study. npj Prim. Care Respir. Med..

[12] Escobedo-Monge M.F., Bahillo-Curieses P., Parodi-Román J., Escobedo-Monge M.A., Alonso-López P., Marugán-Miguelsanz J.M. (2024). Calcium, Phosphate, and Vitamin D in Children and Adolescents with Chronic Diseases: A Cross-Sectional Study. Nutrients.

[13] Sîrbe C., Rednic S., Grama A., Pop T.L. (2022). An Update on the Effects of Vitamin D on the Immune System and Autoimmune Diseases. Int. J. Mol. Sci..

[14] Bikle D.D. (2022). Vitamin D Regulation of Immune Function. Curr. Osteoporos. Rep..

[15] Sanlier N., Guney-Coskun M. (2022). Vitamin D, the immune system, and its relationship with diseases. Egypt. Pediatr. Assoc. Gaz..

[16] Biesalski H.K. (2016). Nutrition meets the microbiome: Micronutrients and the microbiota. Ann. N. Y. Acad. Sci..

[17] Clark A., Mach N. (2016). Role of Vitamin D in the Hygiene Hypothesis: The Interplay between Vitamin D, Vitamin D Receptors, Gut Microbiota, and Immune Response. Front. Immunol..

[18] Zisi D., Challa A., Makis A. (2019). The association between vitamin D status and infectious diseases of the respiratory system in infancy and childhood. Hormones.

[19] McNally J.D., Leis K., Matheson L.A., Karuananyake C., Sankaran K., Rosenberg A.M. (2009). Vitamin D deficiency in young children with severe acute lower respiratory infection. Pediatr. Pulmonol..

[20] Jat K.R. (2017). Vitamin D deficiency and lower respiratory tract infections in children: A systematic review and meta-analysis of observational studies. Trop. Dr..

[21] O’Sullivan M., Moran C., Griffin T.P., Doheny H., McCartney D.M.A., O’Shea P.M. (2024). Impact of the COVID-19 lockdown on the vitamin D status of people in the West of Ireland. Ir. J. Med. Sci..

[22] Qiao Y., Wang X., Ma Y., Hu J. (2025). Variations in vitamin D status among Chinese children aged 1–6 years during the COVID-19 pandemic. Front. Public Health.

[23] Choi J., Choe Y., Lee K., Kim N., Yang S. (2024). Effects of the COVID-19 pandemic on serum vitamin D concentration in Korean children. Ann. Pediatr. Endocrinol. Metab..

[24] Kang C.K., Shin H.M., Park W.B., Kim H.-R. (2022). Why are children less affected than adults by severe acute respiratory syndrome coronavirus 2 infection?. Cell Mol. Immunol..

[25] Luo C., Chen W., Cai J., He Y. (2024). The mechanisms of milder clinical symptoms of COVID-19 in children compared to adults. Ital. J. Pediatr..

[26] Lehmann B. (2005). The Vitamin D_3_ Pathway in Human Skin and its Role for Regulation of Biological Processes. Photochem. Photobiol..

[27] Holick M.F. (2014). Sunlight, ultraviolet radiation, vitamin D and skin cancer: How much sunlight do we need?. Adv. Exp. Med. Biol..

[28] Tugrul B., Demirdag H.G., Hanli Sahin A. (2023). Vitamin D Levels in Children During Winter and the Relationship Between Sunscreen and Sun Protection Behaviors. Dermatol. Pract. Concept..

[29] Nouri N., Iravani P., Abtahi-Naeini B. (2023). Sun protection behaviors among children aged 6−18 years old, the role of socioeconomic factors: A cross-sectional study. Health Sci. Rep..

[30] Neale R.E., Khan S.R., Lucas R.M., Waterhouse M., Whiteman D.C., Olsen C.M. (2019). The effect of sunscreen on vitamin D: A review. Br. J. Dermatol..

[31] Kallioğlu M.A., Sharma A., Kallioğlu A., Kumar S., Khargotra R., Singh T. (2024). UV index-based model for predicting synthesis of (pre-)vitamin D3 in the mediterranean basin. Sci. Rep..

[32] Raymond-Lezman J.R., Riskin S.I. (2023). Benefits and Risks of Sun Exposure to Maintain Adequate Vitamin D Levels. Cureus.

[33] Ginde A.A., Liu M.C., Camargo C.A. (2009). Demographic Differences and Trends of Vitamin D Insufficiency in the US Population, 1988-2004. Arch. Intern. Med..

[34] Lips P., De Jongh R.T., Van Schoor N.M. (2021). Trends in Vitamin D Status Around the World. JBMR Plus.

[35] Basatemur E., Horsfall L., Marston L., Rait G., Sutcliffe A. (2017). Trends in the Diagnosis of Vitamin D Deficiency. Pediatrics.

[36] LeFevre M.L., LeFevre N.M. (2018). Vitamin D Screening and Supplementation in Community-Dwelling Adults: Common Questions and Answers. Am. Fam. Physician.

[37] Krist A.H., Davidson K.W., Mangione C.M., Cabana M., Caughey A.B., Davis E.M., Donahue K.E., Doubeni C.A., Epling J.W., US Preventive Services Task Force (2021). Screening for Vitamin D Deficiency in Adults: US Preventive Services Task Force Recommendation Statement. J. Am. Med. Assoc..

[38] Pourshahidi L.K. (2015). Vitamin D and obesity: Current perspectives and future directions. Proc. Nutr. Soc..

[39] Cashman K.D. (2022). Global differences in vitamin D status and dietary intake: A review of the data. Endocr. Connect..

[40] Alexandru A., Ivan C.-S., Tanasescu S., Oprisoni L.A., Dragomir T.-L., Varga N.-I., Mateescu D., Diaconu M., Margan M.-M., Boeriu E. (2024). Are Pediatric Cancer Patients a Risk Group for Vitamin D Deficiency? A Systematic Review. Cancers.

[41] Nakagawa Y., Koizumi M., Fukagawa M. (2015). Current Topics on Vitamin D. Vitamin D and chronic kidney disease. Clin. Calcium.

[42] Lu C., Yang J., Yu W., Li D., Xiang Z., Lin Y., Yu C. (2015). Association between 25(OH)D Level, Ultraviolet Exposure, Geographical Location, and Inflammatory Bowel Disease Activity: A Systematic Review and Meta-Analysis. PLoS ONE.

[43] Sablok A., Batra A., Thariani K., Batra A., Bharti R., Aggarwal A.R., Kabi B.C., Chellani H. (2015). Supplementation of vitamin D in pregnancy and its correlation with feto-maternal outcome. Clin. Endocrinol..

[44] Zenkert-Andersson K., Hedeland H., Manhem P. (1996). Too little exposure to sun may cause vitamin D deficiency. Muslim women in Sweden are a risk group. Lakartidningen.

[45] Rodda C.P., Benson J.E., Vincent A.J., Whitehead C.L., Polykov A., Vollenhoven B. (2015). Maternal vitamin D supplementation during pregnancy prevents vitamin D deficiency in the newborn: An open-label randomized controlled trial. Clin. Endocrinol..

[46] Parva N.R., Tadepalli S., Singh P., Qian A., Joshi R., Kandala H., Nookala V.K., Cheriyath P. (2018). Prevalence of Vitamin D Deficiency and Associated Risk Factors in the US Population (2011–2012). Cureus.

[47] Raulio S., Erlund I., Männistö S., Sarlio-Lähteenkorva S., Sundvall J., Tapanainen H., Vartiainen E., Virtanen S.M. (2017). Successful nutrition policy: Improvement of vitamin D intake and status in Finnish adults over the last decade. Eur. J. Public Health.

[48] Jääskeläinen T., Itkonen S.T., Lundqvist A., Erkkola M., Koskela T., Lakkala K., Dowling K.G., Hull G.L., Kröger H., Karppinen J. (2017). The positive impact of general vitamin D food fortification policy on vitamin D status in a representative adult Finnish population: Evidence from an 11-y follow-up based on standardized 25-hydroxyvitamin D data. Am. J. Clin. Nutr..

[49] Lehtonen-Veromaa M., Möttönen T., Leino A., Heinonen O.J., Rautava E., Viikari J. (2008). Prospective study on food fortification with vitamin D among adolescent females in Finland: Minor effects. Br. J. Nutr..

[50] Laaksi I.T., Ruohola J.-P.S., Ylikomi T.J., Auvinen A., Haataja R.I., Pihlajamäki H.K., Tuohimaa P.J. (2006). Vitamin D fortification as public health policy: Significant improvement in vitamin D status in young Finnish men. Eur. J. Clin. Nutr..

[51] Piirainen T., Laitinen K., Isolauri E. (2007). Impact of national fortification of fluid milks and margarines with vitamin D on dietary intake and serum 25-hydroxyvitamin D concentration in 4-year-old children. Eur. J. Clin. Nutr..

[52] Pietinen P., Männistö S., Valsta L.M., Sarlio-Lähteenkorva S. (2010). Nutrition policy in Finland. Public Health Nutr..

[53] Cashman K.D., Dowling K.G., Škrabáková Z., Gonzalez-Gross M., Valtueña J., De Henauw S., Moreno L., Damsgaard C.T., Michaelsen K.F., Mølgaard C. (2016). Vitamin D deficiency in Europe: Pandemic?. Am. J. Clin. Nutr..

[54] Brîndușe L.A., Eclemea I., Neculau A.E., Cucu M.A. (2024). Vitamin D Status in the Adult Population of Romania—Results of the European Health Examination Survey. Nutrients.

[55] Nanu M., Stativa E., Ardeleanu I., Moldovanu I., Delia C., Novak I., Stemate M. (2024). Evaluarea Stării de Sănătate COPII—EHES.

[56] WHO Child and Adolescent Health in Europe Report on Progress to 2021. https://www.who.int/europe/publications/i/item/9789289058407.

[57] Herdea A., Marie H., Ionescu A., Sandu D.-M., Pribeagu S.-T., Ulici A. (2024). Vitamin D Deficiency—A Public Health Issue in Children. Children.

[58] Ghiga G., Țarcă E., Țarcă V., Spoială E.L., Păduraru G., Gimiga N., Boca L.O., Iftinchi O., Donos M.A., Manole L.M. (2024). Vitamin D Deficiency: Insights and Perspectives from a Five-Year Retrospective Analysis of Children from Northeastern Romania. Nutrients.

[59] Badiu Tișa I., Cozma-Petruț A., Samașca G., Miere D., Filip L., Banc R., Mîrza O., Iancu M. (2024). Vitamin D Status among 2–18-Year-Old Romanian Pediatric Patients: A Single-Center Study. Nutrients.

[60] Mihu A.G., Nicolescu C.M., Marc C.C., Boru C., Susan M., Ciceu A., Sprintar S.A., Olariu A.T., Oatis D.A., Nicolescu L.C. (2025). Retrospective Serologic Assessment of Vitamin D Levels in Children from Western Romania: A Cross-Sectional Study. Medicina.

[61] Tanase E., Marusca L.M., Horhat F.G., Susan M., Susan R., Horhat R., Dinu S., Dragomir T.-L., Tanasescu S. (2024). Assessing the Impact of Vitamin D Supplementation on Respiratory Infections in Children and Adolescents: A Cross-Sectional Study. Nutrients.

[62] Braegger C., Campoy C., Colomb V., Decsi T., Domellof M., Fewtrell M., Hojsak I., Mihatsch W., Molgaard C., Shamir R. (2013). Vitamin D in the Healthy European Paediatric Population. J. Pediatr. Gastroenterol. Nutr..

[63] Wechsung K., Schnabel D., Wiegand S. (2024). Longitudinal analysis of vitamin D levels considering sunshine duration and suggestion for a standardised approach for vitamin D supplementation in children and adolescents with obesity. BMC Pediatr..

[64] Lapatsanis D., Moulas A., Cholevas V., Soukakos P., Papadopoulou Z.L., Challa A. (2005). Vitamin D: A Necessity for Children and Adolescents in Greece. Calcif. Tissue Int..

[65] Vierucci F., Del Pistoia M., Fanos M., Gori M., Carlone G., Erba P., Massimetti G., Federico G., Saggese G. (2013). Vitamin D status and predictors of hypovitaminosis D in Italian children and adolescents: A cross-sectional study. Eur. J. Pediatr..

[66] Laurent N., Favrais G., Dupont C., Ginies H. (2025). Evaluation of vitamin D supplementation for children under 16 years of age in France. A cross-sectional observational study. Arch. Pediatr..

[67] Sandmann A., Amling M., Barvencik F., König H.-H., Bleibler F. (2017). Economic evaluation of vitamin D and calcium food fortification for fracture prevention in Germany. Public Health Nutr..

[68] Itkonen S.T., Andersen R., Björk A.K., Brugård Konde Å., Eneroth H., Erkkola M., Holvik K., Madar A.A., Meyer H.E., Tetens I. (2021). Vitamin D status and current policies to achieve adequate vitamin D intake in the Nordic countries. Scand. J. Public Health.

[69] Pechabrier M.L., Bacchetta J., Tounian P., Eddiry S., Linglart A., Edouard T. (2025). Survey on vitamin D supplementation in children in France: Evaluation of real-life practices following the new 2022 French recommendations. Arch. Pediatr..

[70] Pop T.L. (2020). Overview of the pediatric healthcare system in Romania. Turk. Arch. Pediatr..

[71] von Elm E., Altman D.G., Egger M., Pocock S.J., Gøtzsche P.C., Vandenbroucke J.P., STROBE Initiative (2008). The Strengthening the Reporting of Observational Studies in Epidemiology (STROBE) statement: Guidelines for reporting observational studies. J. Clin. Epidemiol..

[72] Misra M., Pacaud D., Petryk A., Collett-Solberg P.F., Kappy M., Drug and Therapeutics Committee of the Lawson Wilkins Pediatric Endocrine Society (2008). Vitamin D Deficiency in Children and Its Management: Review of Current Knowledge and Recommendations. Pediatrics.

[73] Grossman Z., Hadjipanayis A., Stiris T., Del Torso S., Mercier J.-C., Valiulis A., Shamir R. (2017). Vitamin D in European children—Statement from the European Academy of Paediatrics (EAP). Eur. J. Pediatr..

[74] Fina S., Heider B., Raţ C. (2021). România Inegală, Disparităţile Socio-Economice Regionale din România.

[75] Dobrow M.J., Hagens V., Chafe R., Sullivan T., Rabeneck L. (2018). Consolidated principles for screening based on a systematic review and consensus process. Can. Med. Assoc. J..

[76] Degiorgio S., Grech N., Dimech Y.M., Xuereb J., Grech V. (2021). Significant Reduction in Pediatric, Population-Based Hospital Admissions Due to COVID-19 in Malta. Turk. Arch. Pediatr..

[77] Zhu Y., Li W., Yang B., Qian R., Wu F., He X., Zhu Q., Liu J., Ni Y., Wang J. (2021). Epidemiological and virological characteristics of respiratory tract infections in children during COVID-19 outbreak. BMC Pediatr..

[78] Miron V.D., Gunșahin D., Filimon C., Bar G., Craiu M. (2022). Pediatric Emergencies and Hospital Admissions in the First Six Months of the COVID-19 Pandemic in a Tertiary Children’s Hospital in Romania. Children.

[79] Gavish R., Levinsky Y., Dizitzer Y., Bilavsky E., Livni G., Pirogovsky A., Scheuerman O., Krause I. (2021). The COVID-19 pandemic dramatically reduced admissions of children with and without chronic conditions to general paediatric wards. Acta Paediatr..

[80] França U.L., McManus M.L. (2024). US Pediatric Inpatient Care Loss Before and During the COVID-19 Pandemic. JAMA Netw. Open.

[81] Fericean R.M., Rosca O., Citu C., Manolescu D., Bloanca V., Toma A.-O., Boeriu E., Dumitru C., Ravulapalli M., Barbos V. (2022). COVID-19 Clinical Features and Outcomes in Elderly Patients During Six Pandemic Waves. J. Clin. Med..

[82] Xue P., Han X., Elahi E., Zhao Y., Wang X. (2021). Internet Access and Nutritional Intake: Evidence from Rural China. Nutrients.

[83] Mosca C., Colucci A., Savoia F., Calì C., Del Bene M., Ranucci G., Maglione A., Pepe A., Morelli A., Vajro P. (2023). Vitamin D Levels in the Pre- and Post-COVID-19 Pandemic Periods and Related Confinement at Pediatric Age. Nutrients.

[84] Yu L., Ke H.-J., Che D., Luo S.-L., Guo Y., Wu J.-L. (2020). Effect of Pandemic-Related Confinement on Vitamin D Status Among Children Aged 0–6 Years in Guangzhou, China: A Cross-Sectional Study. Risk Manag. Healthc. Policy.

[85] Banajeh S.M. (2009). Nutritional rickets and vitamin D deficiency—Association with the outcomes of childhood very severe pneumonia: A prospective cohort study. Pediatr. Pulmonol..

[86] Piloya T., Odongkara B., Were E.M., Ameda F., Mworozi E., Laigong P. (2018). Nutritional rickets among children admitted with severe pneumonia at Mulago hospital, Uganda: A cross-sectional study. BMC Pediatr..

[87] Muhe L., Lulseged S., Mason K.E., Simoes E.A. (1997). Case-control study of the role of nutritional rickets in the risk of developing pneumonia in Ethiopian children. Lancet.

[88] Beyazgül G., Bağ Ö., Yurtseven İ., Coşkunol F., Başer S., Çiçek D., Kanberoğlu G.İ., Çelik F., Nalbantoğlu Ö., Özkan B. (2022). How Vitamin D Levels of Children Changed During COVID-19 Pandemic: A Comparison of Pre-pandemic and Pandemic Periods. J. Clin. Res. Pediatr. Endocrinol..

[89] Lippi G., Henry B.M., Bovo C., Sanchis-Gomar F. (2020). Health risks and potential remedies during prolonged lockdowns for coronavirus disease 2019 (COVID-19). Diagnosis.

[90] Larkin A., Lassetter J. (2014). Vitamin D Deficiency and Acute Lower Respiratory Infections in Children Younger Than 5 Years: Identification and Treatment. J. Pediatr. Health Care.

[91] Alemu E., Varnam R. (2012). Awareness of vitamin D deficiency among at-risk patients. BMC Res. Notes.

[92] Hussein A.S., Almoudi M.M., Mohamed Zen S.A.N., Azmi N.H., Schroth R.J., Abu Hassan M.I. (2018). Parental awareness and knowledge of vitamin D and its health benefits for children. J. Int. Dent. Med. Res..

[93] Toma A.-O., Boeriu E., Decean L., Bloanca V., Bratosin F., Levai M.C., Vasamsetti N.G., Alambaram S., Oprisoni A.L., Miutescu B. (2023). The Effects of Lack of Awareness in Age-Related Quality of Life, Coping with Stress, and Depression Among Patients with Malignant Melanoma. Curr. Oncol..

[94] Alamoudi L.H., Almuteeri R.Z., Al-Otaibi M.E., Alshaer D.A., Fatani S.K., Alghamdi M.M., Safdar O.Y. (2019). Awareness of Vitamin D Deficiency among the General Population in Jeddah, Saudi Arabia. J. Nutr. Metab..

[95] Fitzgerald J.S., Swanson B.J., Larson-Meyer D.E. (2023). Vitamin D Knowledge, Awareness, and Attitudes of Adolescents and Adults: A Systematic Review. J. Nutr. Educ. Behav..

[96] Benskin L.L. (2020). A Basic Review of the Preliminary Evidence That COVID-19 Risk and Severity Is Increased in Vitamin D Deficiency. Front. Public Health.

[97] Ilie P.C., Stefanescu S., Smith L. (2020). The role of vitamin D in the prevention of coronavirus disease 2019 infection and mortality. Aging Clin. Exp. Res..

[98] Entrenas Castillo M., Entrenas Costa L.M., Vaquero Barrios J.M., Alcalá Díaz J.F., López Miranda J., Bouillon R., Quesada Gomez J.M. (2020). Effect of calcifediol treatment and best available therapy versus best available therapy on intensive care unit admission and mortality among patients hospitalized for COVID-19: A pilot randomized clinical study. J. Steroid Biochem. Mol. Biol..

[99] McKenna M.J., Lyons O.C., Flynn M.A., Crowley R.K., Twomey P.J., Kilbane M.T. (2022). COVID-19 pandemic and vitamin D: Rising trends in status and in daily amounts of vitamin D provided by supplements. BMJ Open.

[100] Heer R.S., Sandhu P., Wenban C., Mandal A.K.J., Missouris C.G. (2022). Vitamin D in the news: A call for clear public health messaging during COVID-19. Nutr. Health.

[101] Alibrahim H., Swed S., Bohsas H., Abouainain Y., Jawish N., Diab R., Ishak A., Saleh H.H., Nasif M.N., Arafah R. (2024). Assessment the awareness of vitamin D deficiency among the general population in Syria: An online cross-sectional study. BMC Public Health.

[102] Al-Mutairi N., Nair V., Issa B. (2012). Photoprotection and vitamin D status: A study on awareness, knowledge and attitude towards sun protection in general population from Kuwait, and its relation with vitamin D levels. Indian J. Dermatol. Venereol. Leprol..

[103] Blebil A.Q., Dujaili J.A., Teoh E., Wong P.S., Kc B. (2019). Assessment of Awareness, Knowledge, Attitude, and the Practice of Vitamin D among the General Public in Malaysia. J. Karnali Acad. Health Sci..

[104] Caillet P., Goyer-Joos A., Viprey M., Schott A.-M. (2017). Increase of vitamin D assays prescriptions and associated factors: A population-based cohort study. Sci. Rep..

[105] Assche S.B.-V., Ferraccioli F., Riccetti N., Gomez-Ramirez J., Ghio D., Stilianakis N.I. (2024). Urban-rural disparities in COVID-19 hospitalisations and mortality: A population-based study on national surveillance data from Germany and Italy. PLoS ONE.

[106] Zulfakhar Zubir M., Aizuddin A.N., Abdul Manaf M.R., Aziz Harith A., Ihsanuddin Abas M., Izyami Kayat M., Radi M.F.M., Norehan Merican M., Fitra N., Ali A.M. (2025). Three decades in healthcare service efficiency evaluation: A bootstrapping Data Envelopment Analysis (DEA) of Ministry of Health Malaysia. Health Econ. Rev..

[107] Vogiatzi M.G., Jacobson-Dickman E., DeBoer M.D., Drugs, and Therapeutics Committee of the Pediatric Endocrine Society (2014). Vitamin D Supplementation and Risk of Toxicity in Pediatrics: A Review of Current Literature. J. Clin. Endocrinol. Metab..

[108] Levita J., Wilar G., Wahyuni I., Bawono L.C., Ramadaini T., Rohani R., Diantini A. (2023). Clinical Toxicology of Vitamin D in Pediatrics: A Review and Case Reports. Toxics.

[109] Farnaghi F., Hassanian-Moghaddam H., Zamani N., Gholami N., Gachkar L., Hosseini Yazdi M. (2020). Vitamin D toxicity in a pediatric toxicological referral center; a cross-sectional study from Iran. BMC Pediatr..

[110] United Nations Development Programme (2016). Human Development Report 2016: Human Development for Everyone.

[111] Kahwati L.C., LeBlanc E., Weber R.P., Tanner R.M., Viswanathan E.K., Feltner C.J., Jonas D.E. (2021). Screening for Vitamin D Deficiency in Adults: An Evidence Review for the U.S. Preventive Services Task Force.

